# Mutations in the α4-α5 allosteric lobe of RAS do not significantly impair RAS signaling or self-association

**DOI:** 10.1016/j.jbc.2022.102661

**Published:** 2022-11-09

**Authors:** Michael Whaby, Lauren Wallon, Megan Mazzei, Imran Khan, Kai Wen Teng, Shohei Koide, John P. O’Bryan

**Affiliations:** 1 Department of Cell and Molecular Pharmacology & Experimental Therapeutics, Medical University of South Carolina, Charleston, South Carolina, USA; 2 Hollings Cancer Center, Medical University of South Carolina, Charleston, South Carolina, USA; 3 Department of Biochemistry and Molecular Pharmacology, New York University Grossman School of Medicine, New York, New York, USA; 4 Ralph H. Johnson VA Medical Center, Charleston, South Carolina, USA; 5 Perlmutter Cancer Center, New York University Langone Health, New York, New York, USA

**Keywords:** GTPase, dimerization, ERK-MAPK, nanoclustering, oncogenesis, FBS, fetal bovine serum, GAP, GTPase activating protein, HVR, hypervariable region, MAPK, mitogen-activated protein kinase, PEI, polyethylenimine, PPI, protein–protein interaction

## Abstract

Mutations in one of the three *RAS* genes (*HRAS*, *KRAS*, and *NRAS*) are present in nearly 20% of all human cancers. These mutations shift RAS to the GTP-loaded active state due to impairment in the intrinsic GTPase activity and disruption of GAP-mediated GTP hydrolysis, resulting in constitutive activation of effectors such as RAF. Because activation of RAF involves dimerization, RAS dimerization has been proposed as an important step in RAS-mediated activation of effectors. The α4-α5 allosteric lobe of RAS has been proposed as a RAS dimerization interface. Indeed, the NS1 monobody, which binds the α4-α5 region within the RAS G domain, inhibits RAS-dependent signaling and transformation as well as RAS nanoclustering at the plasma membrane. Although these results are consistent with a model in which the G domain dimerizes through the α4-α5 region, the isolated G domain of RAS lacks intrinsic dimerization capacity. Furthermore, prior studies analyzing α4-α5 point mutations have reported mixed effects on RAS function. Here, we evaluated the activity of a panel of single amino acid substitutions in the α4-α5 region implicated in RAS dimerization. We found that these proposed “dimerization-disrupting” mutations do not significantly impair self-association, signaling, or transformation of oncogenic RAS. These results are consistent with a model in which activated RAS protomers cluster in close proximity to promote the dimerization of their associated effector proteins (*e.g.*, RAF) without physically associating into dimers mediated by specific molecular interactions. Our findings suggest the need for a nonconventional approach to developing therapeutics targeting the α4-α5 region.

## Teaser

Mutations of residues implicated for RAS dimerization do not impair oncogenic RAS signaling.

RAS GTPases are important mediators of intracellular signaling cascades that regulate cell proliferation and survival (1, 2). The three *RAS* genes (*HRAS, KRAS,* and *NRAS*) encode four different protein isoforms: HRAS, splice variants KRAS4A and KRAS4B, and NRAS. Each of the RAS isoforms cycle through active (RAS-GTP) and inactive (RAS-GDP and apoRAS) states that are tightly regulated by GTPase activating proteins (GAPs) and guanine nucleotide exchange factors (3). Prenylation and palmitoylation of cysteine residues of the C-terminal hypervariable region (HVR) of RAS localize it to the inner leaflet of the cell membrane where RAS is typically activated in response to growth factor receptor–mediated stimulation (4). However, activating mutations of RAS stabilize the active RAS-GTP state even in the absence of upstream stimulation (1). Notably, nearly 20% of all human cancers harbor such RAS mutations, making RAS the most frequently mutated oncogene as well as a valuable target for cancer therapy (5).

Clinically available inhibitors of RAS have been elusive. However, the recent FDA approval of the KRAS^G12C^ inhibitor, sotorasib, illustrates the feasibility of pharmacological inhibition of KRAS (6, 7). Unfortunately, KRAS^G12C^ represents only a fraction of oncogenic RAS mutations in human cancers (8, 9), presenting a critical need for alternative RAS inhibitors. Due to the picomolar affinity of RAS for guanine nucleotides, targeting the nucleotide binding pocket has been considered pharmacologically problematic (10), although recent work provides support for the possibility of targeting the nucleotide-free state of RAS (11). In the absence of direct RAS inhibitors, interfering with RAS membrane localization has been explored as an alternative therapeutic approach (12). However, the ability of KRAS to be alternatively prenylated in response to farnesyl transferase inhibitor treatment rendered this approach ineffective for KRAS-mutant cancers (13), although these inhibitors have shown some efficacy in HRAS-mutant malignancies (14).

More recent studies have highlighted the possibility of inhibiting RAS nanoclustering as a potential approach to inhibit RAS-mediated signaling and biological activity both *in vitro* and *in vivo* (15, 16, 17, 18, 19). Utilizing monobody technology (20), we previously reported that the NS1 monobody specifically binds the α4-α5 allosteric lobe of HRAS and KRAS, but not NRAS, and inhibits RAS dimers/nanoclusters (15). Additionally, the K13 and K19 DARPins inhibit KRAS via binding of the α3-α4 allosteric lobe and disrupting RAS dimer/nanoclusters (17). The importance of RAS dimers/nanoclusters was further highlighted by the endogenous RAS antagonist, DIRAS3, which inhibits RAS nanoclusters through binding of α5 region of RAS (21). These results are consistent with the reported presence of dimers of RAS in a number of crystal structures of RAS (15, 22, 23, 24), the GTP-dependent dimerization of the KRAS G domain (25), and the observation that artificial dimerization of the isolated G domain of RAS is sufficient to activate RAF *in vitro* (26) and increase mitogen-activated protein kinase (MAPK) signaling in cells (27). In contrast, disruption of these higher-order RAS assemblies inhibits downstream signaling and biological function *in vivo* (15, 16, 17, 18, 19).

Although these examples implicate RAS dimerization as an on-pathway event in RAS-mediated signaling and provide a rationale for drug discovery efforts aimed at development of RAS dimerization inhibitors, the importance of RAS dimerization remains a point of much debate. While active RAS stimulates the dimerization and activation of RAF kinases in the MAPK pathway (28), the G domain of HRAS lacks the propensity to dimerize in solution (23). Furthermore, the isolated HVR of KRAS, devoid of the G domain, is sufficient to drive dimerization of a fluorescent protein–HVR fusion protein in cells, suggesting that KRAS G domain is dispensable for dimerization (27). In light of these studies, many attempts have been made to identify binding interfaces that facilitate higher-order RAS assemblies. Three major RAS dimerization interfaces have been proposed: the α3-α4 interface, the α4-α5 interface, and the β-sheet interface (29). Experimental evidence supporting the validity of these dimerization interfaces, however, has been conflicting. Although RAS was inhibited by the NS1 monobody, point mutations within the NS1-binding interface of HRAS predicted to disrupt dimerization (*e.g.*, R135A, D154Q, R161D) did not impair downstream MAPK pathway activation (15). Similarly, a recent study reported that the KRAS^D154Q^ mutation did not affect RAS–RAS interaction in cells (30). In contrast, Ambrogio *et al.* provided evidence that mutation of D154Q or R161E decreased KRAS association (as measured by FRET), CRAF–BRAF association, and KRAS-mediated signaling and tumor formation *in vivo* (24). More recent work has suggested that RAF RBD engagement promotes RAS dimerization through the α4-α5 region although mutations in this region did not affect RAS dimerization (31).

Because of the contradictory results with these studies, we sought to characterize a panel of oncogenic HRAS, KRAS, and NRAS mutants to assess the effects of α4-α5 mutations on oncogenic RAS function. These mutations were selected based on previous reports of their importance in RAS dimer formation (15, 22, 25, 32, 33, 34). Here, we show that mutations in the α4-α5 allosteric lobe do not affect RAS association in cells. In addition, these mutants did not impair MAPK activation compared with the parental oncogenic RAS. Although downstream signaling was unaffected by these mutations, we observed isoform-specific differences in the transforming activity of selected mutants. Overall, our results are consistent with a model in which RAS protomers associate in close proximity to promote effector dimerization (*e.g.*, RAF) without formation of molecularly defined RAS dimers. Further, we propose that the ability of selected biologics such as NS1 monobody and K13/K19 DARPins to perturb RAS function is due to steric hinderance of RAS rather than disruption of *bona fide* dimers. This study provides further insights into RAS oligomerization and should inform efforts in developing therapeutics directed at the α4-α5 allosteric lobe of RAS.

## Results

### Mutations in the α4-α5 region of KRAS do not impair oncogenic activity

**Figure 1:**
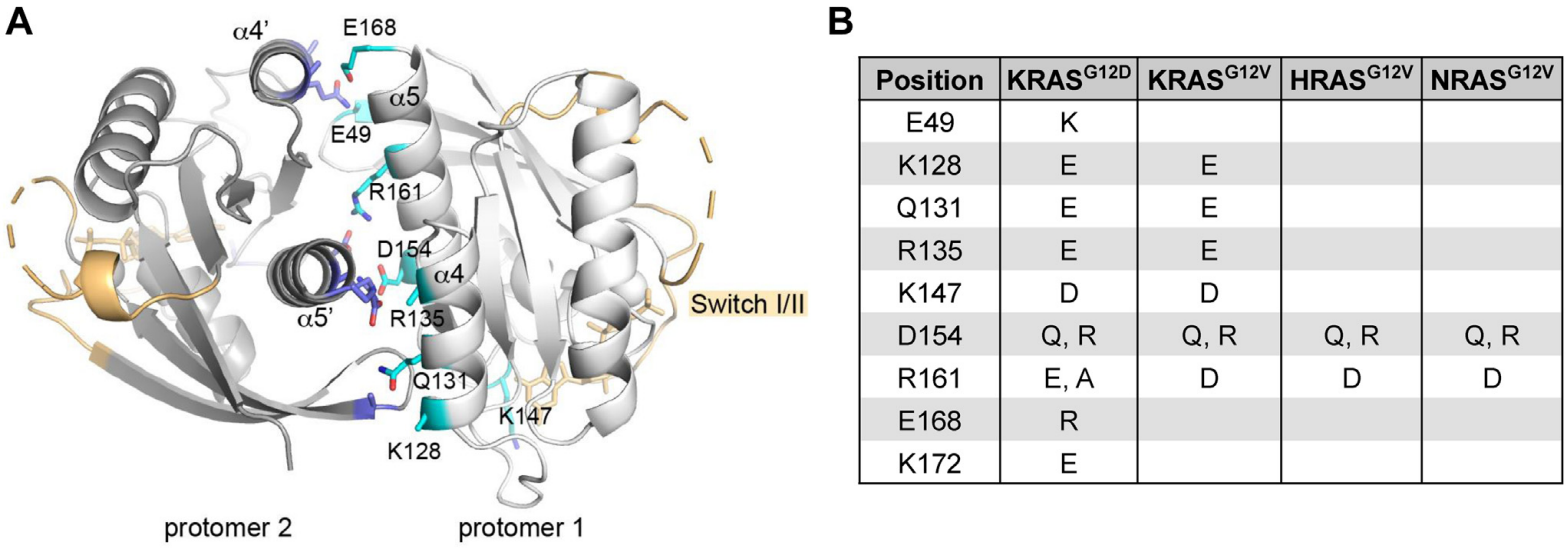
**A model of an α4-α5 dimer of KRAS and the locations of residues tested in this study.***A*, The model was built by fitting the structure of KRAS4B•GMPPNP (residues 1–169; PDB ID 6VC8; (49)) onto the crystallographic dimer of HRAS•GMPPNP (residues 1–166; PDB ID 5P21; (50)). The Switch I and II regions are colored in *tan*. The α4 and α5 helices in protomer 1 are labeled, and those in protomer 2, that is, the symmetry-related copy, are labeled as α4′ and α5′. The residues subjected to mutational studies are shown in *cyan* and labeled for protomer 1 and shown in *blue* for protomer 2. The side chains of E49, K128, and R135 are disordered in the 6VC8 model and thus not depicted. *B*, RAS α4-α5 mutants included in this study.

**Figure 2:**
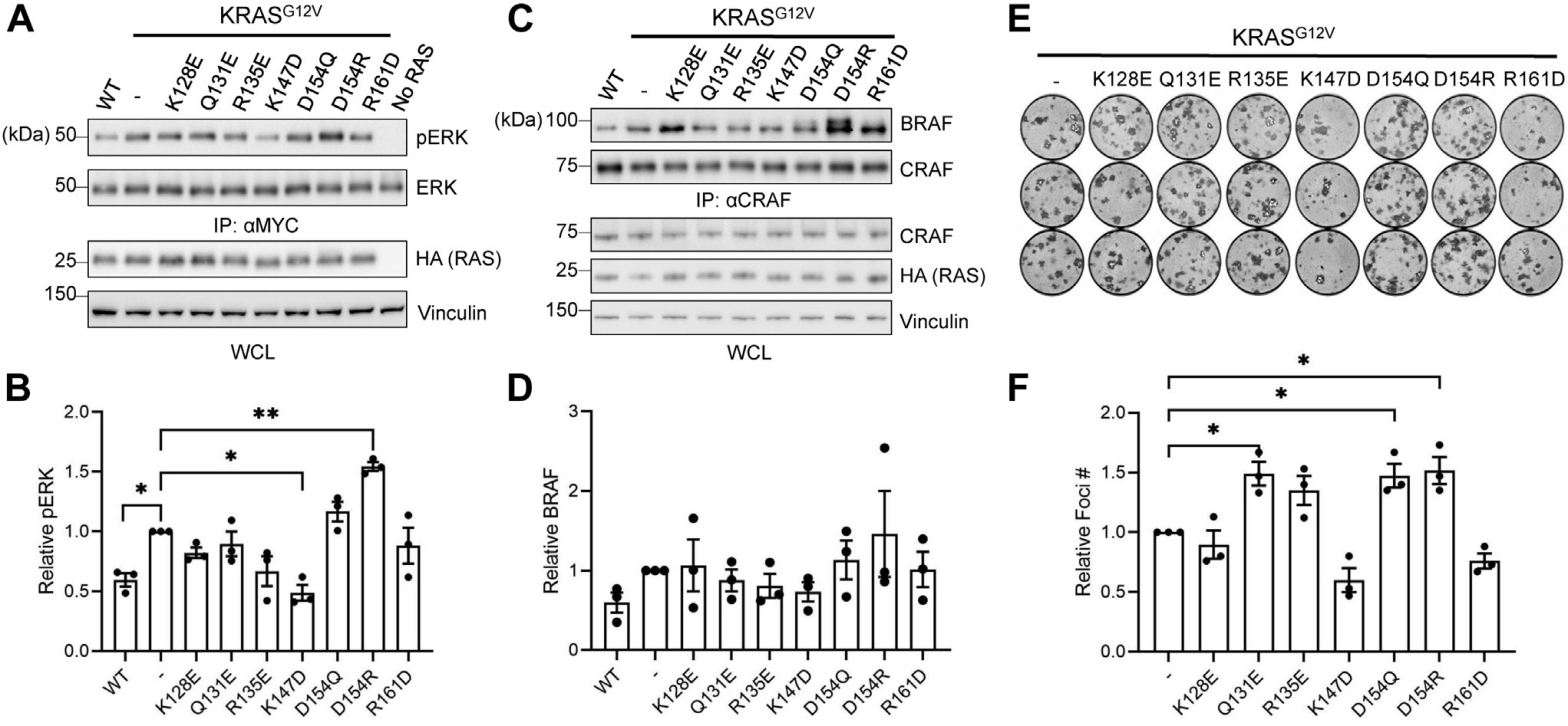
**Mutations in the α4-α5 allosteric lobe of KRAS do not impair oncogenic activity.***A*, ERK/MAPK activity assay in HEK 293 cells cotransfected with HA-tagged KRAS^G12V^ mutants and MYC-tagged ERK. *B*, Normalized pERK signal from panel (*A*). *C*, Immunoprecipitation (IP) of endogenous CRAF in HEK 293 cells transfected with KRAS^G12V^ mutants. *D*, Normalized BRAF signal from the CRAF IP in panel (*C*). *E*, NIH/3T3 transformation assay in cells transfected with KRAS^G12V^ mutants. *F*, Normalized foci number from the NIH/3T3 transformation assays in panel (*E*). All experiments were repeated at least three times (n = 3) and results quantified using Welch’s *t* test; error bars representing SEM (∗∗∗*p* < 0.0005, ∗∗*p* < 0.005, and ∗*p* < 0.05). MAPK, mitogen-activated protein kinase.

Given the contradictory reports on the importance of dimerization in RAS function, we tested whether mutations in the proposed dimerization interface (Fig. 1, *A* and *B*; termed α4-α5 mutants hereafter) impaired MAPK pathway activation relative to the parental oncogenic KRAS mutant. To reduce overexpression artifacts, we determined the appropriate amounts of K/H/NRAS DNA that yielded protein expression and ERK phosphorylation below the point of saturation for the signaling assays (Figs. S1–S4). Surprisingly, there were no significant differences in MAPK activation between the KRAS^G12V^ α4-α5 mutants and the parental oncogenic KRAS^G12V^ with the exception of KRAS^G12V/K147D^, a mutation that has been shown to decrease RAS-GTP levels (Figs. 2, *A* and *B*, and S5) (35, 36).

Next, we analyzed CRAF–BRAF association in cells expressing KRAS^G12V^ *versus* KRAS^G12V^ harboring α4-α5 mutants, given the well-established role of RAF dimerization in MAPK signaling (37, 38). In addition, a previous study demonstrated that D154Q and R161D KRAS mutants decreased CRAF–BRAF heterodimers (24). In agreement with the results from the MAPK signaling assays described above, there was no significant impairment in CRAF–BRAF interaction in cells expressing the KRAS α4-α5 mutants compared with parental KRAS^G12V^ (Fig. 2, *C* and *D*). These data indicate that the α4-α5 mutations do not impair the activation of the canonical RAS/MAPK pathway mediated by KRAS^G12V^.

Lastly, we performed transformation assays in NIH/3T3 cells transfected with KRAS^G12V^ or the KRAS^G12V^ α4-α5 mutants (Fig. 2, *E* and *F*). Consistent with the signaling data, the KRAS^G12V^ α4-α5 mutants retained the ability to transform cells as well as parental KRAS^G12V^. Taken together, these results suggest that mutations of amino acid residues proposed to be critical for KRAS–KRAS self-association do not significantly impair the signaling or transforming properties of oncogenic KRAS.

### Mutations in the α4-α5 allosteric lobe do not affect KRAS association in cells

Next, we addressed whether these α4-α5 mutations affected RAS–RAS association in cells. We employed Live-Cell NanoLuc Binary Technology (NanoBiT), a protein–protein interaction (PPI) detection system where one protein partner is tagged with an 11-amino acid peptide (SmBiT), while the other protein partner is tagged with a 17.6 kDa NanoLuc fragment (LgBiT). When expressed in cells, PPIs between the two protein partners allows for complementation of the SmBiT and LgBiT tags to generate a luminescent signal upon substrate addition.

**Figure 3:**
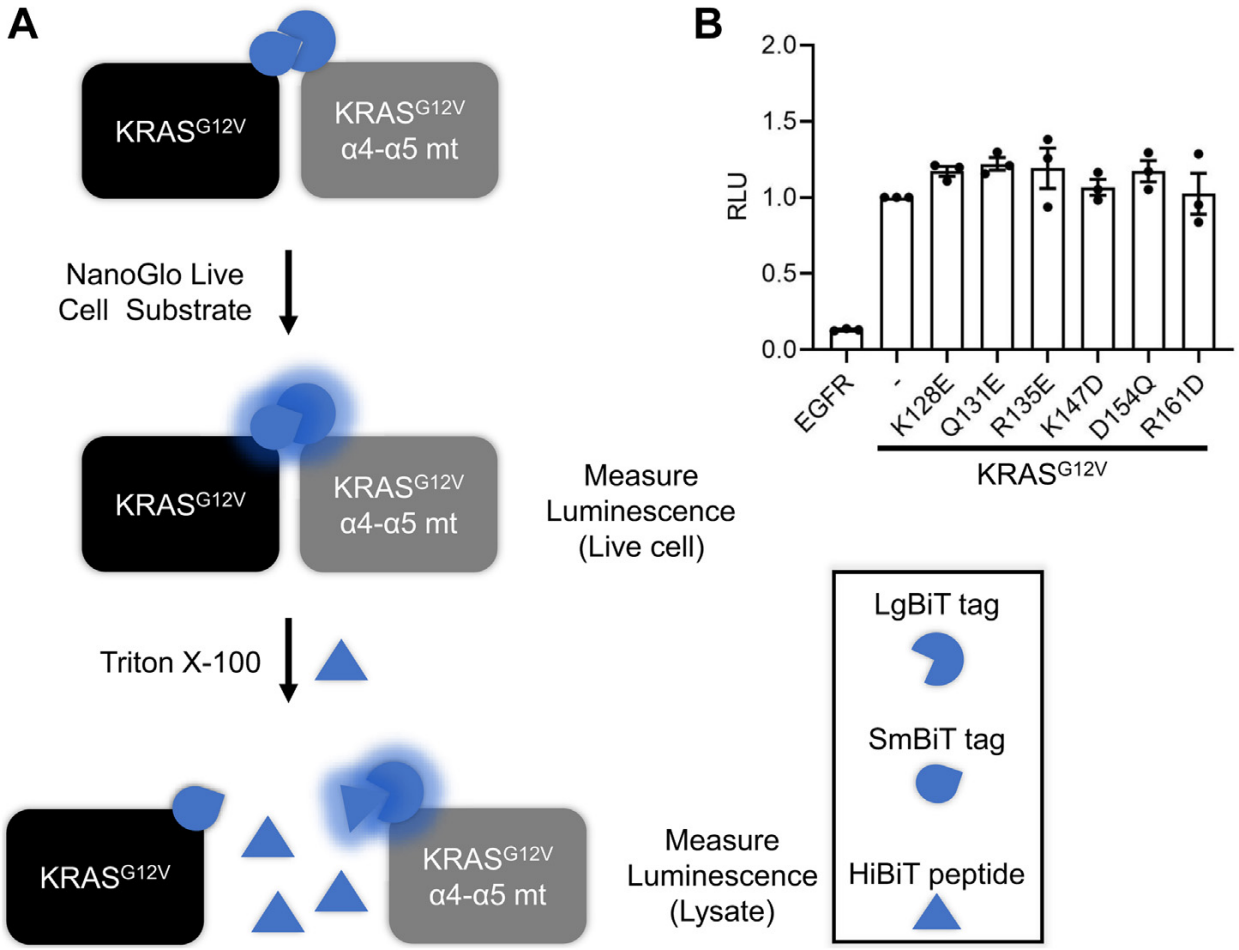
**Mutations in the α4-α5 allosteric lobe of RAS do not affect RAS–RAS association in cells.***A*, Illustration of the workflow for the NanoBiT assay used to measure PPIs in live cells. *B*, NanoBiT assay in RAS-less MEFs (BRAF^V600E^) coexpressing SmBiT-KRAS^G12V^ and LgBiT-KRAS^G12V^, LgBiT-KRAS^G12V^ α4-α5 mutants, or EGFR-LgBiT. All experiments were repeated at least three times (n = 3) and results quantified using Welch’s *t* test; error bars representing SEM (∗∗∗*p* < 0.0005, ∗∗*p* < 0.005, and ∗*p* < 0.05). PPI, protein–protein interaction.

To avoid potential interference of endogenous RAS proteins with the SmBiT/LgBiT-tagged KRAS protein partners, we performed these assays in RAS-less MEFs transformed with BRAF^V600E^, a cell line that lacks all 3 RAS isoforms (39). To account for variations between transfections, cells were lysed after measuring the live-cell luminescence (Fig. S8*A*), and LgBiT-tagged proteins were quantified using HiBiT, an 11 amino acid peptide with high affinity (K_D_ = 0.7 nM) for the LgBiT peptide. The HiBiT peptide out-competes the SmBiT peptide for LgBiT binding while still generating luminescent signal, allowing for a fast and sensitive method to quantify total LgBiT peptide levels in cells (Figs. 3*A* and S8*B*). When coexpressed in cells, SmBiT-KRAS^G12V^ and LgBiT-KRAS^G12V^ reconstituted luciferase activity (Fig. 3, *A* and *B*). In contrast, coexpression of SmBiT-KRAS^G12V^ with EGFR-LgBiT, also a membrane-localized protein which served as a negative control, generated a weak luminescent signal (Fig. 3*B*). The introduction of the α4-α5 mutations to LgBiT-KRAS^G12V^ resulted in no significant decreases in luciferase activity (Fig. 3*B*). These results are consistent with the results from signaling assays (Fig. 2), indicating that not only do the α4-α5 mutations have little to no effect on KRAS signaling and biology, they also do not impair RAS–RAS interactions in cells.

### RAS α4-α5 mutants are still susceptible to inhibition by the NS1 monobody

**Figure 4:**
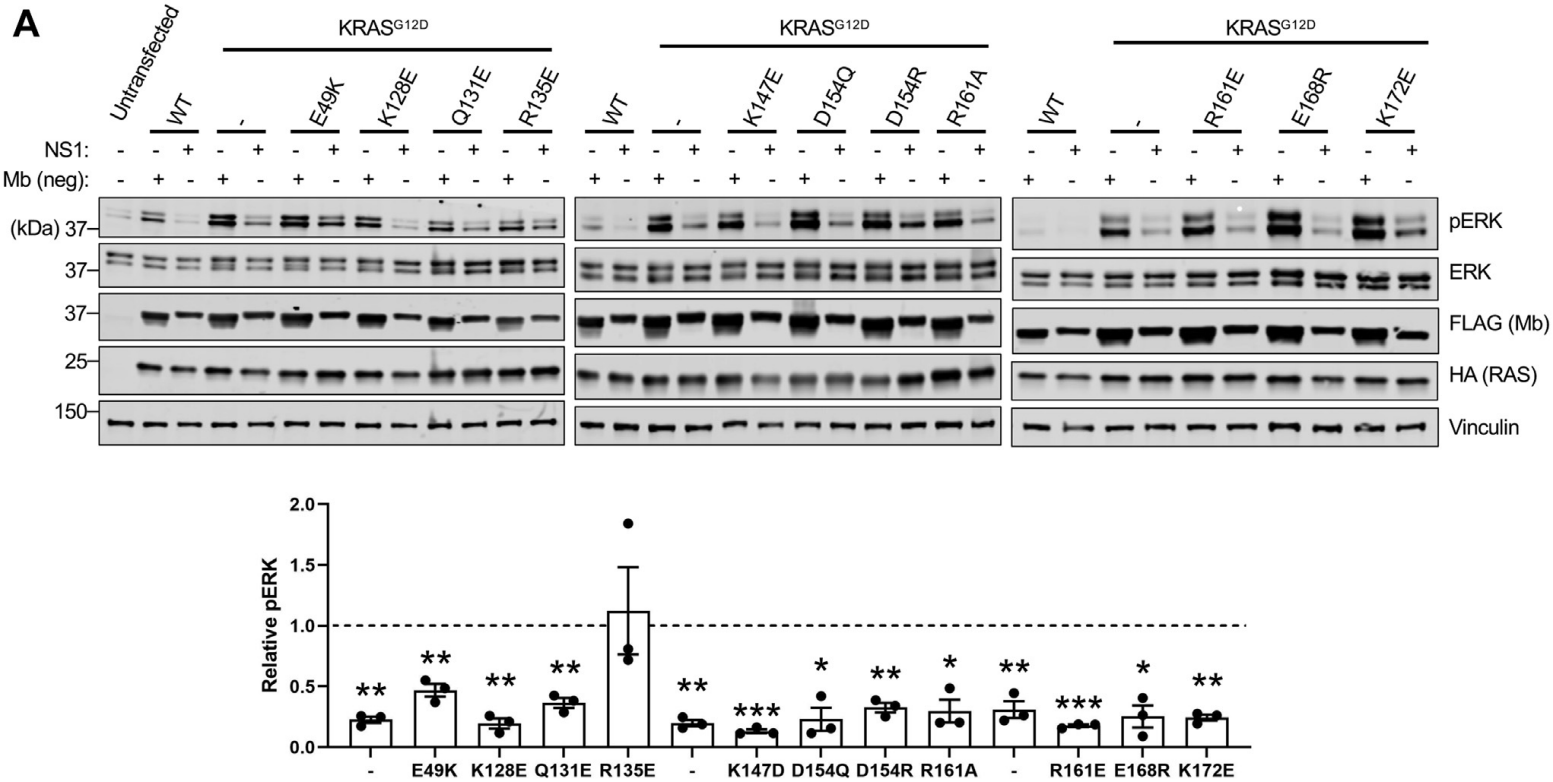
**RAS α4-α5 mutants are still susceptible to inhibition by the NS1 monobody.***A*, ERK/MAPK signaling assay in HEK 293 cells cotransfected with indicated HA-tagged KRAS^G12D^ mutants and FLAG-tagged NS1 or a negative control monobody (Mb (neg)). Results are representative of one of three biological replicates. Graph represents the relative pERK levels. The mean and sd (n = 3) for the normalized pERK/ERK in the NS1 compared to Mb (neg) sample is shown. *Dotted line* at 1 represent pERK levels in Mb (neg) samples. All *p* values were generated using an unpaired Student’s *t* test (∗∗∗*p* < 0.0005, ∗∗*p* < 0.005, and ∗*p* < 0.05). MAPK, mitogen-activated protein kinase.

NS1 inhibits oncogenic RAS-mediated signaling, biological transformation, and tumor formation (15, 18, 19) and disrupts higher-order RAS associations at the plasma membrane (15). Through binding of the α4-α5 region of RAS, NS1 allosterically inhibits RAS function irrespective of its nucleotide state (15, 16). We hypothesized that if the α4-α5 mutations decreased RAS–RAS interaction in cells, then NS1 would have less of an inhibitory effect on these mutants compared with the parental RAS oncogenic mutants. As illustrated in Figures S6 and S7, α4-α5 mutations in KRAS^G12D^ did not impact ERK activation. Furthermore, coexpression of NS1 effectively inhibited downstream ERK phosphorylation in cells expressing KRAS^G12D^ and KRAS^G12D^ α4-α5 mutants (Figs. 4*A* and S9). The inhibitory effect of NS1 was highly specific as KRAS^G12D/R135E^, which does not bind NS1 (15), was refractory to inhibition by NS1 (Figs. 4*A* and S9). These results further support the notion that these α4-α5 mutations are not sufficient to impair RAS association or oncogenic RAS-mediated signaling in cells.

### HRAS and NRAS α4-α5 mutants exhibit isoform-specific biological properties

**Figure 5:**
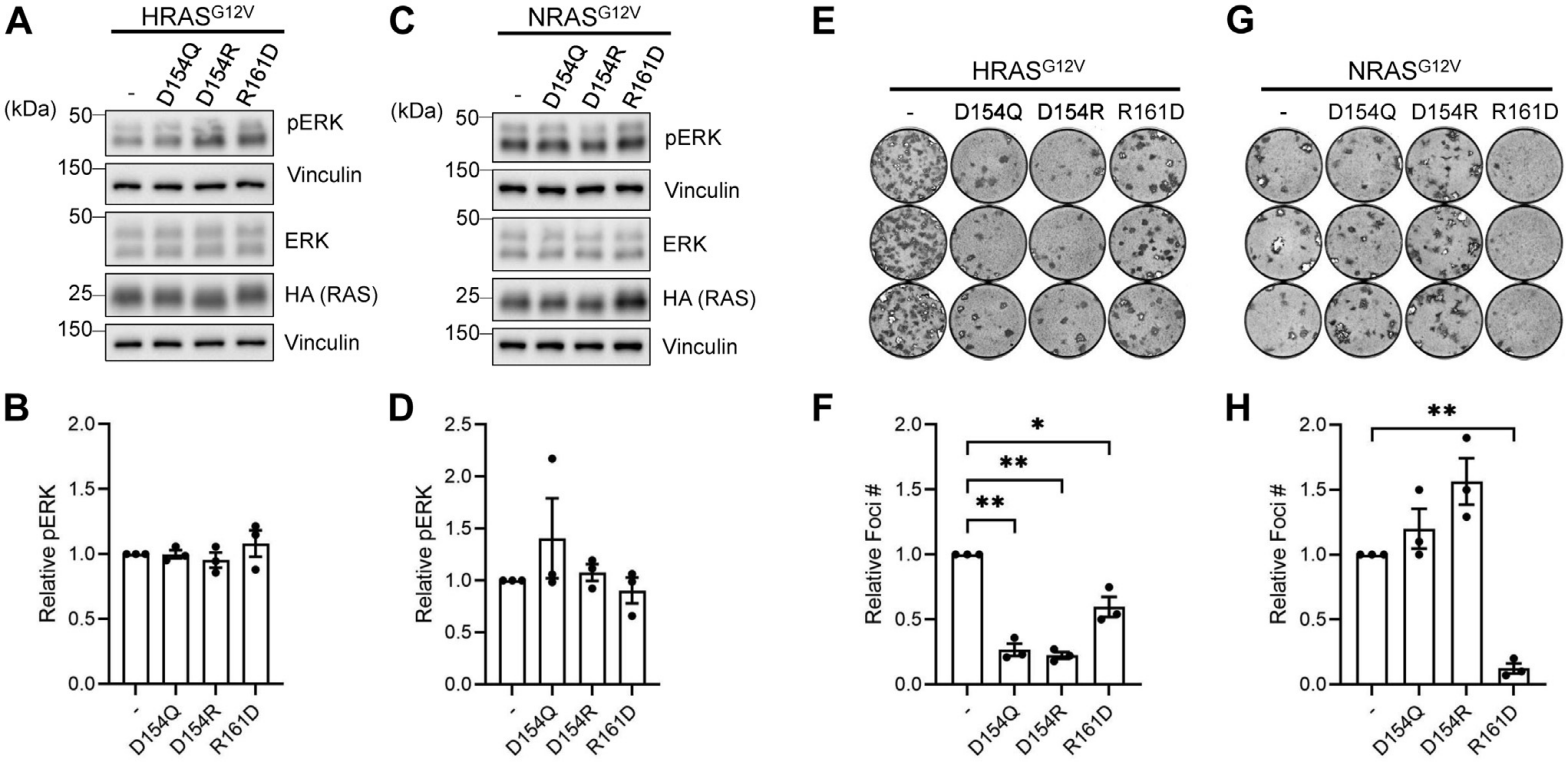
**HRAS and NRAS α4-α5 mutants exhibit isoform-specific biological properties.***A*, ERK/MAPK activity assay in HEK 293 cells transfected with HA-tagged HRAS^G12V^ mutants. *B*, Normalized pERK signal from panel (*A*). *C*, ERK/MAPK activity assay in HEK 293 cells transfected with HA-tagged NRAS^G12V^ mutants. *D*, Normalized pERK signal from panel (*C*). *E*, NIH/3T3 transformation assay in cells transfected with HRAS^G12V^ mutants. *F*, Normalized foci number from the NIH/3T3 transformation assays in panel (*E*). *G*, NIH/3T3 transformation assay in cells transfected with NRAS^G12V^ mutants. *H*, normalized foci number from the NIH/3T3 transformation assays in panel (*E*). All experiments were repeated at least three times (n = 3) and results quantified using Welch’s *t* test; error bars representing SEM (∗∗∗*p* < 0.0005, ∗∗*p* < 0.005, and ∗*p* < 0.05). MAPK, mitogen-activated protein kinase.

We then examined whether α4-α5 mutations in the other RAS isoforms had similar effects on their signaling and biological properties. In contrast to previously reported results with KRAS^D154Q^ (24), we reported that mutations at D154 or R161 of HRAS^G12V^ had no effect on ERK-MAPK activation (15), which might suggest isoform-specific effects of these mutations. Consistent with prior findings (15, 30) and the results with KRAS^G12V^ (Fig. 2), mutations at D154 or R161 had no effect on either HRAS^G12V^ or NRAS^G12V^ signaling (Figs. 5, *A*–*D* and S10).

Next, we assessed the impact of these mutations on the biological activity of H/NRAS^G12V^. Whereas the KRAS^G12V^ mutants showed no significant differences in foci formation (Fig. 2, *E* and *F*), all of the HRAS^G12V^ α4-α5 mutants (Fig. 5, *E* and *F*) and one of the NRAS^G12V^ α4-α5 mutants (R161D) (Fig. 5, *G* and *H*) resulted in significantly fewer foci than those of the parental control. Together, these data show that mutations in the α4-α5 allosteric lobe of all three RAS isoforms have no effect on ERK-MAPK signaling but impair the transforming activity of HRAS^G12V^ and to a lesser extent NRAS^G12V^.

## Discussion

The link between RAS dimerization and RAS-mediated signaling was first observed by Santos *et al.* in 1998 (40) and was revisited at the turn of the 21st century (26). Indeed, artificial dimerization of RAS at the plasma membrane activated the MAPK pathway (27), while inhibition of RAS clusters at the plasma membrane is associated with inhibition of the MAPK pathway (15, 21). Although there is still debate surrounding the exact mechanism of RAS nanoclustering at the plasma membrane, the α4-α5 region of RAS has been proposed in several studies to be an important interface contributing several stabilizing interactions to facilitate formation of RAS dimers (15, 22, 25, 32, 33, 34, 41). Based on these studies, mutations within the α4-α5 allosteric lobe (*e.g.*, D154Q) have been proposed to disrupt the interactions necessary to form higher-order RAS assemblies. However, there has been conflicting data surrounding the ability of these RAS mutants to impact signaling and biology.

Consistent with our previous results (15), we found that single point mutations within the α4-α5 allosteric lobe of RAS predicted to disrupt dimer formation (Fig. 1) did not decrease downstream MAPK pathway activation compared with parental oncogenic RAS, with the exception of the KRAS^G12V/K147D^ mutant. K147 is an important site for posttranslational modifications that regulate RAS activity. Monoubiquitylation of K147 impedes the RAS-GAP interaction and hence GAP-stimulated GTP hydrolysis, thereby favoring the RAS-GTP state (35, 42, 43). Furthermore, K147 acetylation regulates nucleotide binding and is associated with increased KRAS activity and tumor growth *in vivo* (44). Thus, the reduction in downstream MAPK signaling from KRAS^G12V/K147D^, especially since it did not impair self-association of KRAS (Fig. 3), could be a consequence of the K147D mutation preventing these tumor-promoting posttranslational modifications. Consistent with the MAPK signaling activity, there was no significant impairment in foci formation from the KRAS^G12V^ α4-α5 mutants compared with KRAS^G12V^. Together, these results demonstrate that mutations at residues in the α4-α5 region that have been proposed to mediate RAS dimerization do not impair the oncogenic signaling or biological activity of KRAS. This suggests that if these mutations truly disrupted dimerization, then RAS dimerization, per se, may not be a necessary, on-pathway step for oncogenic RAS signaling. Conversely, if RAS dimerization is necessary for its activity, then our data suggest that these mutations do not affect RAS–RAS interactions to the extent necessary to inhibit oncogenic signaling.

This study provides evidence that not only do the KRAS α4-α5 mutants retain the ability to activate MAPK signaling, but they also do not have impaired PPIs with other RAS monomers in cells. While NS1 does not disrupt the association of RAF with HRAS, it decreases CRAF–BRAF association in cells, reflecting the ability of NS1 to sterically interfere with RAS clustering at the plasma membrane (15). In contrast, the KRAS^G12V^ α4-α5 mutants were not significantly impaired in their ability to induce CRAF–BRAF interaction compared with KRAS^G12V^, which was also reflected in the MAPK signaling assays. These results were further corroborated by the NanoBiT assays showing that the α4-α5 mutations did not affect KRAS–KRAS association in cells. Furthermore, these mutants remained sensitive to inhibition by NS1. Overall, we have shown that oncogenic KRAS is essentially unimpaired by mutations in the α4-α5 allosteric lobe.

The isoform-specific differences in transformation observed with the D154 and R161 mutations in HRAS and the R161 mutation in NRAS were unexpected but raise important questions. First, could these mutations disrupt differential effector activation from the RAS isoforms? For instance, Yan *et al.* (1998) reported that KRAS potently activated RAF but was less efficient at activating PI3K when compared with HRAS (45). While there is no evidence for PI3K dimerization, targeting RAS with NS1 monobody decreased downstream phospho-AKT levels (15) suggesting that disrupting RAS nanoclusters may affect multiple pathways. Second, it is possible that each isoform may utilize distinct mechanisms of nanoclustering (41). Nevertheless, given the inability of these α4-α5 mutations to impair oncogenic RAS signaling (*i.e.*, RAF-MAPK activation), we conclude that either these mutations do not affect RAS dimerization or that RAS dimerization is not needed for RAS-induced MAPK activation.

**Figure 6:**
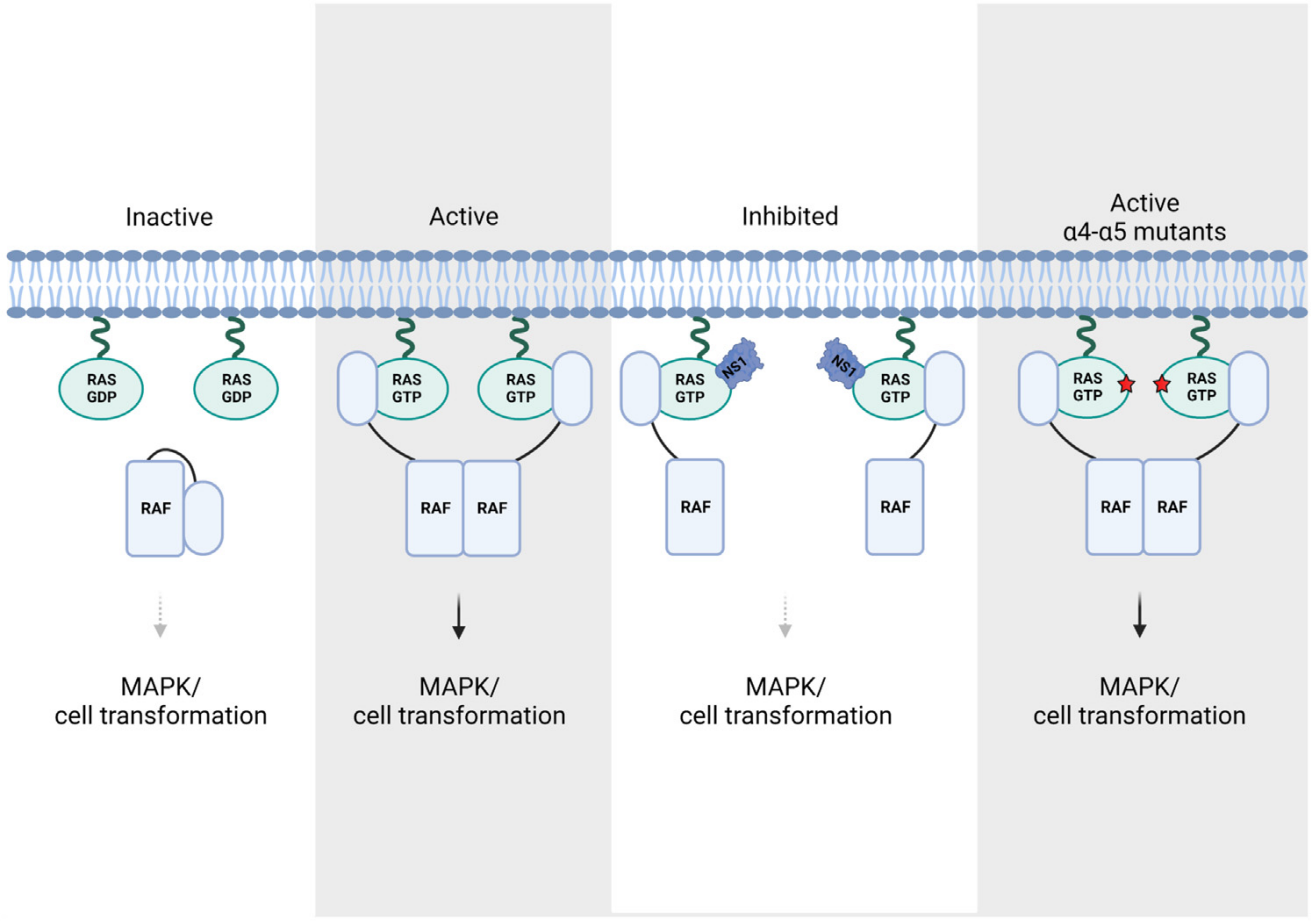
**Model for the role of the α4-α5 region of oncogenic RAS higher-order assembly.** GTP loading of RAS results in recruitment of RAF through binding of the CRD-RBD region of RAF resulting in unmasking of the dimerization interface on RAF. This results in RAF-mediated clustering of activated RAS. NS1 disrupts RAS clusters and RAS signaling through steric hinderance which prevents RAF from forming productive dimers. Mutations within the α4-α5 region are insufficient to disrupt this clustering due to a lack of electrostatic interactions between requisite amino acid side chains. Created with BioRender.com

Our results are consistent with a model in which RAS protomers rely on proximity, but not direct association with one another to form a signaling-competent complex (Fig. 6). The required proximity may be in the form of loosely associated nanoclusters where RAS protomers are close enough to promote RAF dimerization but do not require well-defined interactions between amino acid side chains of residues within the α4-α5 allosteric lobe. Disruption of these nanoclusters may require larger molecules, such as NS1 or DIRAS3, which may reduce the density of RAS on the membrane surface and/or distort the RAS–RAF complex into an inactive conformation (46). In contrast, point mutations of specific amino acid residues on the α4-α5 lobe of RAS do not appear sufficient to disrupt the interactions necessary for downstream pathway activation. The implications of these results for drug design are that smaller molecules targeting the α4-α5 allosteric lobe may be insufficient to impair downstream pathway activation. Instead, an approach to bring a large molecule to the α4-α5 region utilizing “glue” compounds (47) may be required to exploit this vulnerability of RAS.

## Experimental procedures

### Cell culture and cloning

Freshly thawed HEK 293 (MUSC Tissue Culture Facility) and NIH/3T3 (National Institutes of Health) cells were maintained in Dulbecco’s modified Eagle’s medium (DMEM) (Corning) supplemented with 10% fetal bovine serum (FBS) or 10% calf serum, respectively. RAS-less MEFs (BRAF^V600E^) were obtained from the National Cancer Institute and maintained in DMEM supplemented with 10% FBS and 4 μg/ml blasticidin. RAS α4-α5 mutants were generated via site-directed mutagenesis using pCGN-HA-RAS^G12V^ or pCGN-HA-RAS^G12D^ as templates for each isoform. Primers used to generate RAS α4-α5 mutants are listed in Table 1. All monobodies were subcloned into CMV-driven expression vectors containing an mCherry-tag followed by a FLAG-tag on the N-terminus. KRAS^G12V^ was subcloned into a high-expression, CMV-driven vector downstream of the SmBiT cDNA sequence. Similarly, KRAS^G12V^ and the α4-α5 mutants were cloned downstream of LgBiT-containing vectors; however, these vectors were low-expression, HSV-driven vectors. Lastly, EGFR-LgBiT clone was in a high-expression, CMV-driven vector. All NanoBiT vectors were provided by Matt Robers and Dr Jim Vasta (Promega).

**Table 1:** Primers used to generate the RAS^G12V^ α4-α5 mutants analyzed in this study.

	α4–α5 mutation	Forward Primer (5' to 3')	Reverse Primer (5' to 3')
KRAS^G12V^	K128E	CCAGAACAGTAGACACAGAACAGGCTCAGGACTTAGCA	TGCTAAGTCCTGAGCCTGTTCTGTGTCTACTGTTCTGG
	Q131E	CAGTAGACACAAAACAGGCTGAGGACTTAGCAAGAAGT	ACTTCTTGCTAAGTCCTCAGCCTGTTTTGTGTCTACTG
	R135E	CAGGCTCAGGACTTAGCAGAAAGTTATGGAATTCCTTT	AAAGGAATTCCATAACTTTCTGCTAAGTCCTGAGCCTG
	K147D	TTTATTGAAACATCAGCAGACACAAGACAGGGTGTTGA	TCAACACCCTGTCTTGTGTCTGCTGATGTTTCAATAAA
	D154Q	ACAAGACAGGGTGTTGATCAAGCCTTCTATACATTAGTT	AACTAATGTATAGAAGGCTTGATCAACACCCTGTCTTGT
	D154R	ACAAGACAGGGTGTTGATAGAGCCTTCTATACATTAGTT	AACTAATGTATAGAAGGCTCTATCAACACCCTGTCTTGT
	R161D	GCCTTCTATACATTAGTTGATGAAATTCGAAAACATAAA	TTTATGTTTTCGAATTTCATCAACTAATGTATAGAAGGC
HRAS^G12V^	D154Q	ACCCGGCAGGGAGTGGAGCAGGCCTTCTACACGTTGGTG	CACCAACGTGTAGAAGGCCTGCTCCACTCCCTGCCGGGT
	D154R	ACCCGGCAGGGAGTGGAGCGGGCCTTCTACACGTTGGTG	CACCAACGTGTAGAAGGCCCGCTCCACTCCCTGCCGGGT
	R161D	GATGCCTTCTACACGTTGGTGGACGAGATCCGGCAGCAC	GTGCTGCCGGATCTCGTCCACCAACGTGTAGAAGGCATC
NRAS^G12V^	D154Q	ACCAGACAGGGTGTTGAACAAGCTTTTTACACACTGGTA	TACCAGTGTGTAAAAAGCTTGTTCAACACCCTGTCTGGT
	D154R	ACCAGACAGGGTGTTGAAAGAGCTTTTTACACACTGGTA	TACCAGTGTGTAAAAAGCTCTTTCAACACCCTGTCTGGT
	R161D	GCTTTTTACACACTGGTAGATGAAATACGCCAGTACCGA	TCGGTACTGGCGTATTTCATCTACCAGTGTGTAAAAAGC

### Transfections and cell signaling assays

Transfections and cell signaling assays were performed as previously described (11, 48). Briefly, HEK 293 cells were transfected with HA-tagged RAS using polyethylenimine (PEI). Typically, we transfected cells using 3 μl of PEI for every 1 μg of DNA. When indicated, HA-tagged RAS was cotransfected with MYC-tagged ERK for signaling assays. Transfected cells were incubated for 30 h in complete media (DMEM with 10% FBS), then serum-starved overnight. MYC-tagged ERK was immunoprecipitated from the cell lysates using α-MYC antibody (Millipore-Sigma), then ERK and pERK levels were analyzed via Western Blot using α-ERK (Cell Signaling Technology) and α-pERK (Cell Signaling Technology) antibodies. ERK and pERK protein levels were quantified using Image Studio Lite (Ver 5.2) software. pERK/ERK ratio was determined for each mutant and normalized to the parental oncogenic RAS mutant. Each experiment was performed three times (n = 3).

To analyze CRAF–BRAF association, HEK 293 cells were transfected with the indicated RAS mutants using the same conditions as described above. After cell lysates were collected, a coimmunoprecipitation was done by pulling down endogenous CRAF using α-CRAF (BD Biosciences) antibody and probing for CRAF and BRAF via Western Blot with α-CRAF (BD Biosciences) and α-BRAF (Santa Cruz) antibodies. BRAF/CRAF ratio was determined for each mutant and all values were normalized to the parental oncogenic RAS mutant. Each experiment was performed three times (n = 3).

For signaling assays performed with KRAS^G12D^ and the NS1 monobody, 1 × 10^6^ HEK293T cells were cultured 24 h before transfection on a 6-well plate using DMEM supplemented with 10% FBS. Cells that were between 70 and 90% confluent were then serum starved and transfected with either KRAS variants or in a 1:1 ratio with monobodies using lipofectamine according to the manufacturer’s protocol. Raw band intensity was evaluated using Image Studio Lite Version 5.2. For calculating the normalized (pERK/ERK)/HA, each band was first normalized to vinculin. For calculating the NS1/Mb (neg) ratio, pERK/ERK ratio was first calculated then used to generate the NS1/Mb (neg) ratio. Statistical analysis was performed using GraphPad Prism 9.

### Immunoblotting and antibodies

Following experimental endpoints as described above, cell lysates were made by washing cells with PBS followed by addition of PLC lysis buffer (50 mM Hepes, pH 7.5, 150 mM NaCl, 10% glycerol, 1% Triton X-100, 1 mM EGTA, 1.5 mM magnesium chloride, 100 mM sodium fluoride supplemented with 1 mM vanadate, 10 mg/ml leupeptin, and 10mg/ml aprotinin). Lysates were nutated at 4 °C for 1 h, then centrifuged at 14K RPM for 10 min. Supernatant-containing protein was transferred to new tubes and stored at −80 °C. The Pierce BCA Protein Assay Kit (ThermoFisher) was used to quantify protein in cell lysates before Western Blot analyses. The following antibodies were used: monoclonal HA (clone 16B12, Biolegend #90154), monoclonal FLAG (Clone M2, Sigma #F1804), phospho-ERK (Thr202/Tyr204, CST #9101), total ERK (CST #9102), Vinculin (SC #73614), Anti-MYC (Clone A46, Millipore-Sigma #05–724), CRAF (BD Biosciences # 610151), BRAF (Santa Cruz #sc-9002).

### NIH/3T3 transformation assays

For NIH/3T3 transformation assays, 2.5 × 10^5^ cells were split into 60 mm tissue culture plates and seeded overnight. The following day, cells were transfected with the indicated RAS mutants using PEI transfection method (48). Media on cells was changed every 2 days following transfections. Oncogenic RAS induced foci formation approximately 2 to 3 weeks following transfections, and cells were fixed and stained with 0.1% crystal violet before quantification of foci. Assays were performed three times each (n = 3).

### NanoBiT protein–protein interaction assays

For NanoBiT PPI assays, 3.0 × 10^4^ cells per well (RAS-less MEFs) were plated in a white-wall, clear-bottom 96-well plate (Thermo Scientific 165306) and incubated at 37 °C overnight. The next day, all wells were transfected with SmBiT-KRAS^G12V^, and selected wells were transfected (technical replicates per experiment = 6) with either EGFR-LgBiT, LgBiT-KRAS^G12V^, or LgBiT-KRAS^G12V^ α4-α5 mutants using PEI transfection method. Twenty four hours after transfection, media was aspirated from wells, and luminescence was measured using NanoGlo Live-Cell Substrate (Promega; Cat # N2012) suspended in Opti-MEM reduced serum media (Gibco; cat # 31985070). After the live-cell luminescence measurement, cells were lysed with 1.0% Triton X-100 and incubated with HiBiT peptide (0.1 μM) for 10 min on orbital shaker. Then, luminescence was measured to quantify LgBiT peptide levels. Live-cell luminescence was normalized to luminescence after HiBiT peptide addition, and all samples were normalized to SmBiT-KRAS^G12V^/LgBiT-KRAS^G12V^. Assays were performed three times each (n = 3).

### Statistical analysis

All statistical analyses were performed using GraphPad Prism 9 software.

## Data availability

All reagents in this article are available upon request and completion of an MTA with the Medical University of South Carolina and/or New York University.

## Supporting information

This article contains supporting information.

## Conflict of interest

J. P. O., A. K., and S. K. are listed as inventors on a patent application on Monobodies targeting the nucleotide-free state of RAS files by the Medical University of South Carolina and New York University (No. 62/862924). K. W. T., A. K., and S. K. are listed as inventors on a patent application on mutant RAS targeting Monobodies filed by New York University (Application No. 63/121903). A. K. and S. K. are listed as inventors on issued and pending patents on Monobody technology filed by The University of Chicago (US Patent 9512199 B2 and related pending applications). S. K. was an SAB member and holds equity in and received consulting fees from Black Diamond Therapeutics and receives research funding from Black Diamond Therapeutics, Puretech Health, and Argenx BVBA. The other authors declare no competing interests.
