# Supplementary material for: Mutations in the α4-α5 allosteric lobe of RAS do not significantly impair RAS signaling or self-association
Source: J Biol Chem. 2022 Nov 9;298(12):102661. doi: 10.1016/j.jbc.2022.102661 (PMC9763690; doi:10.1016/j.jbc.2022.102661)
Supplement: Supplemental Figures [file mmc1.pdf]

Supplemental Figure 1.

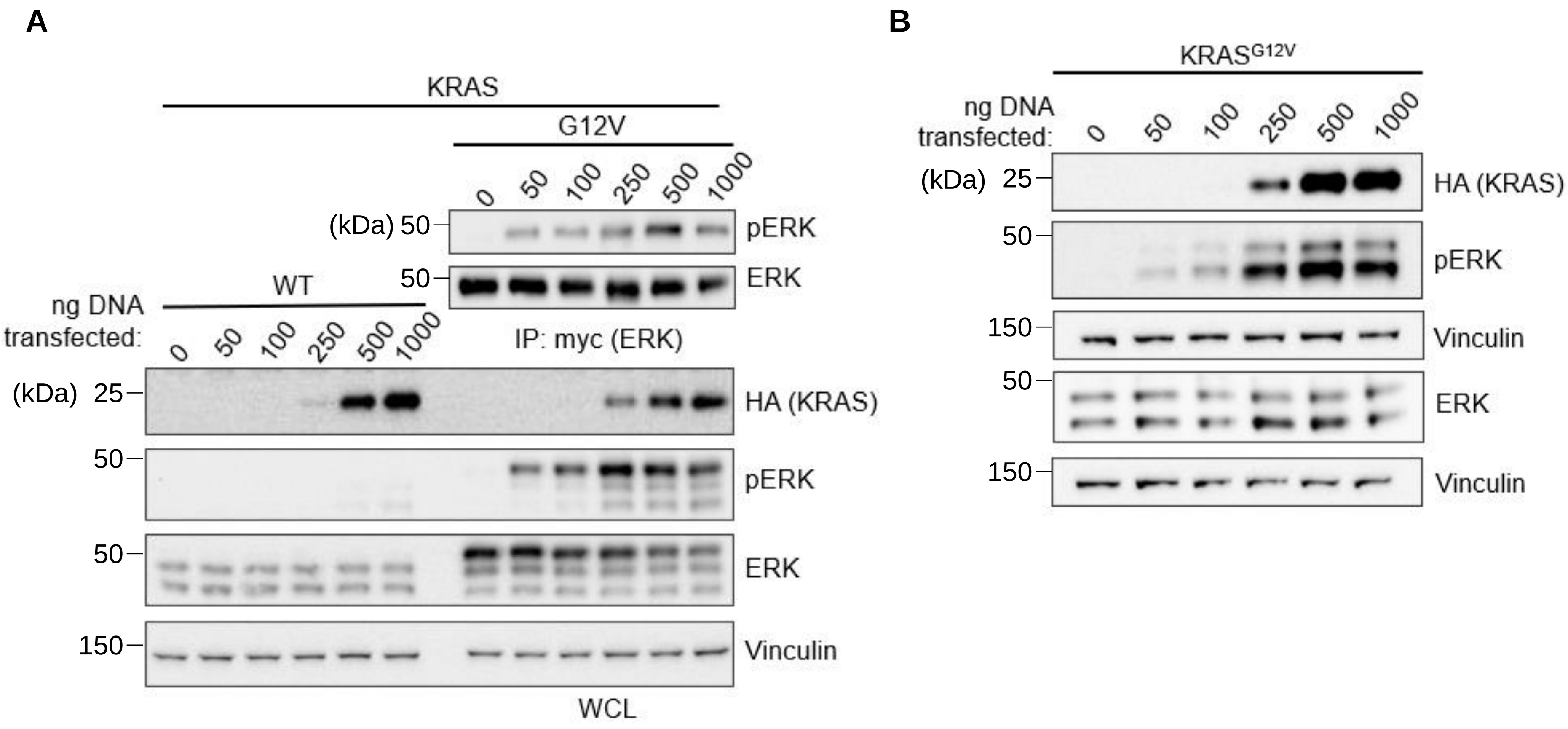

Supplemental Figure 1. A) Titration of pCGN-HA-KRAS WT and G12V in HEK 293 cells. Cells transfected with KRAS G12V were co-transfected with MYC-ERK to analyze pERK signal strictly from transfected cells. B) KRAS G12V titration in HEK 293 cells.

Supplemental Figure 2.

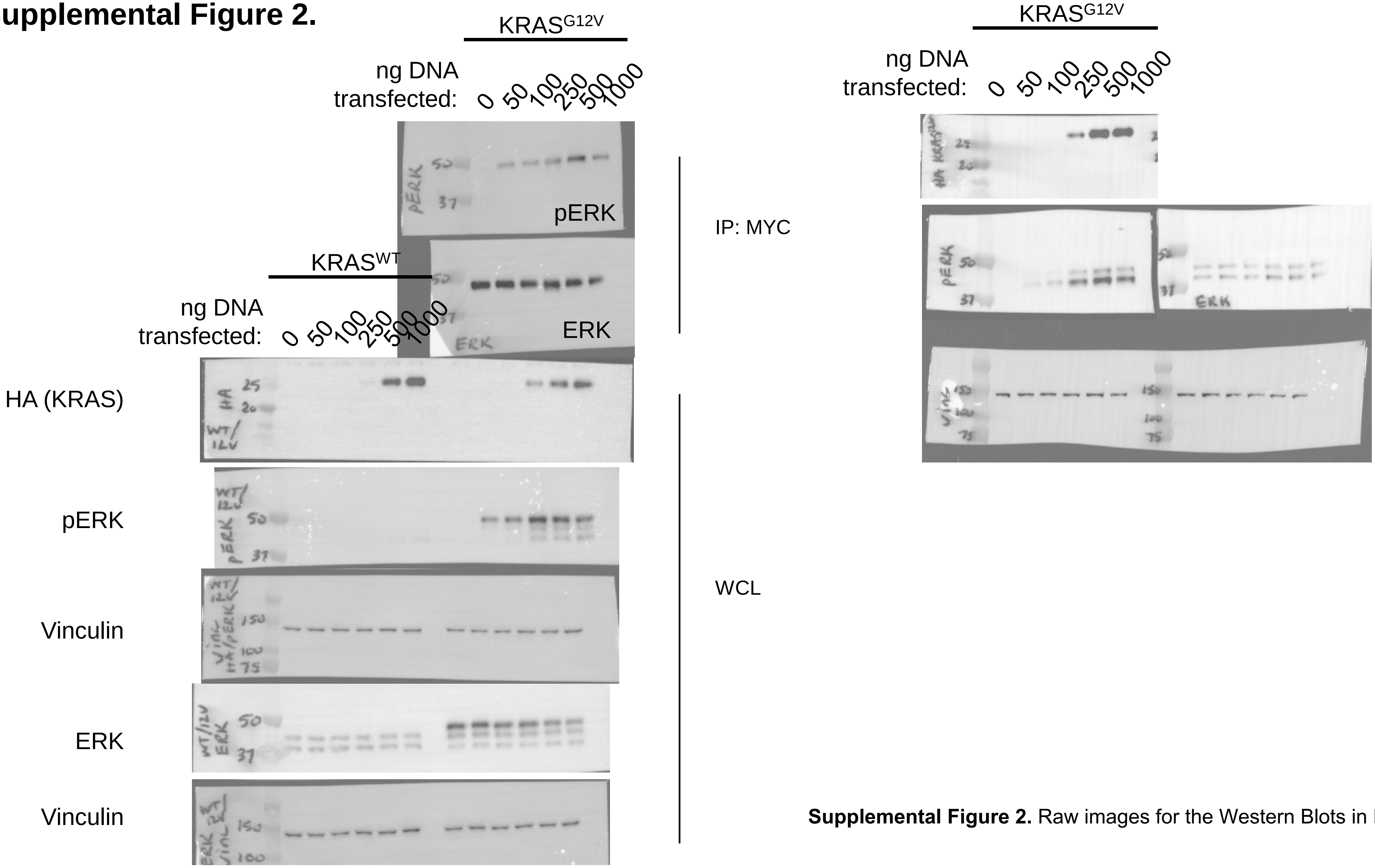

Supplemental Figure 2. Raw images for the Western Blots in Fig. S1.

Supplemental Figure 3.

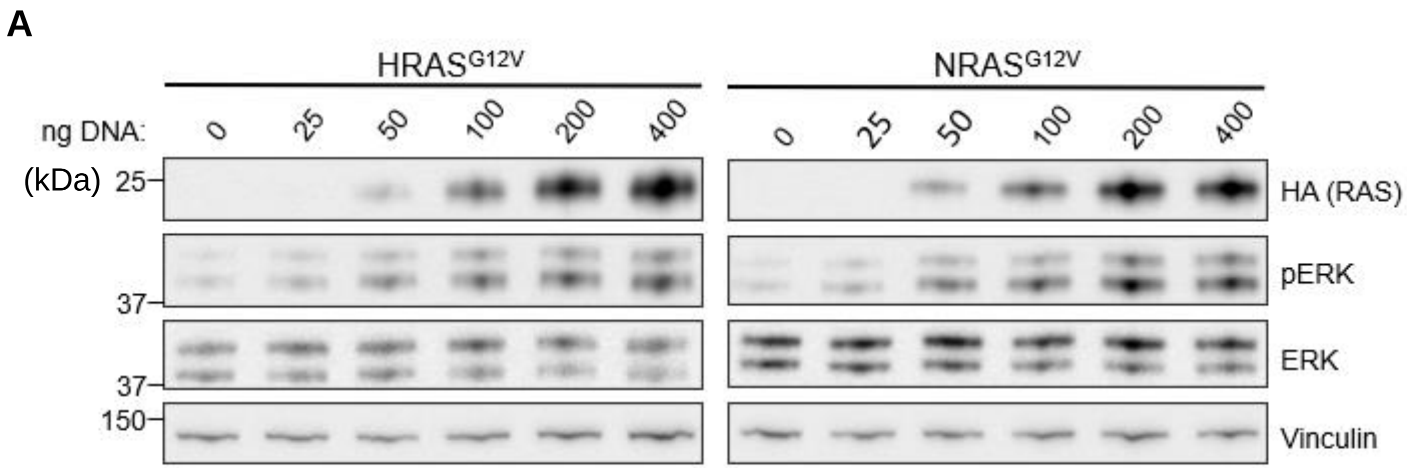

Supplemental Figure 3. Transfection titration of pCGN-HA-HRAS and NRAS G12V in HEK 293 cells.

Supplemental Figure 4.

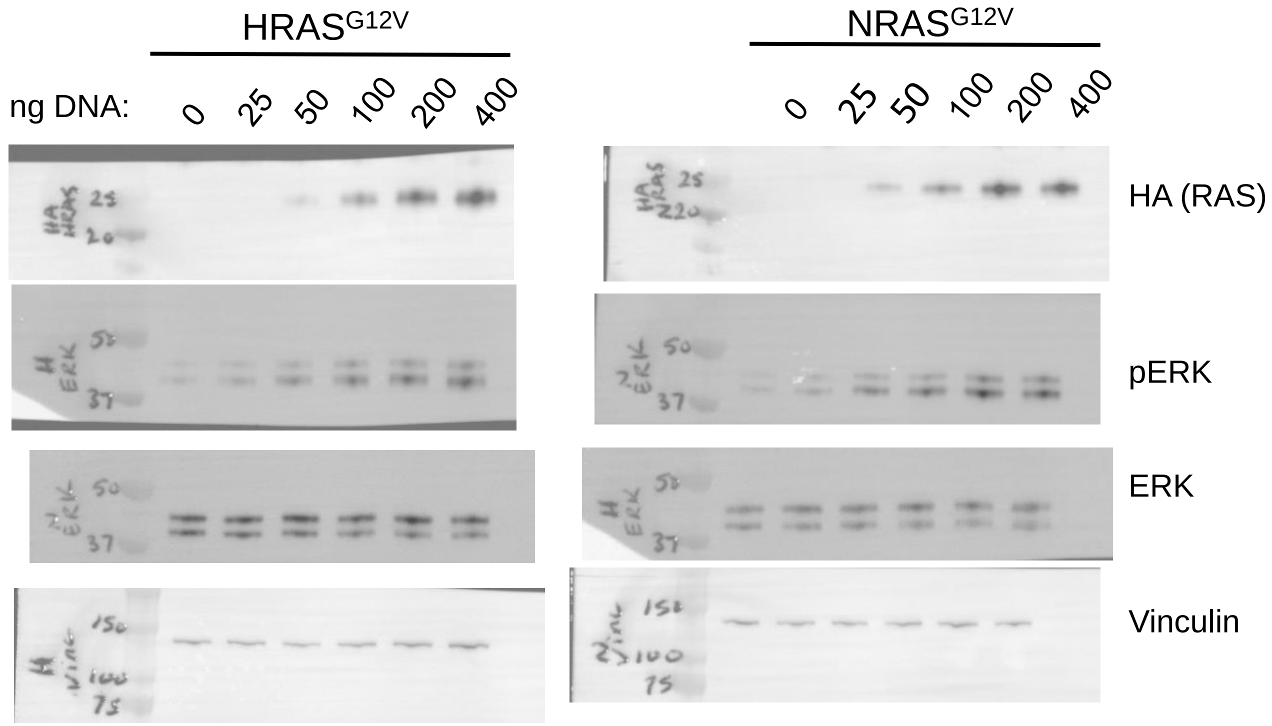

Supplemental Figure 4. Raw images from the Western Blots in Fig. S3.

Supplemental Figure 5.

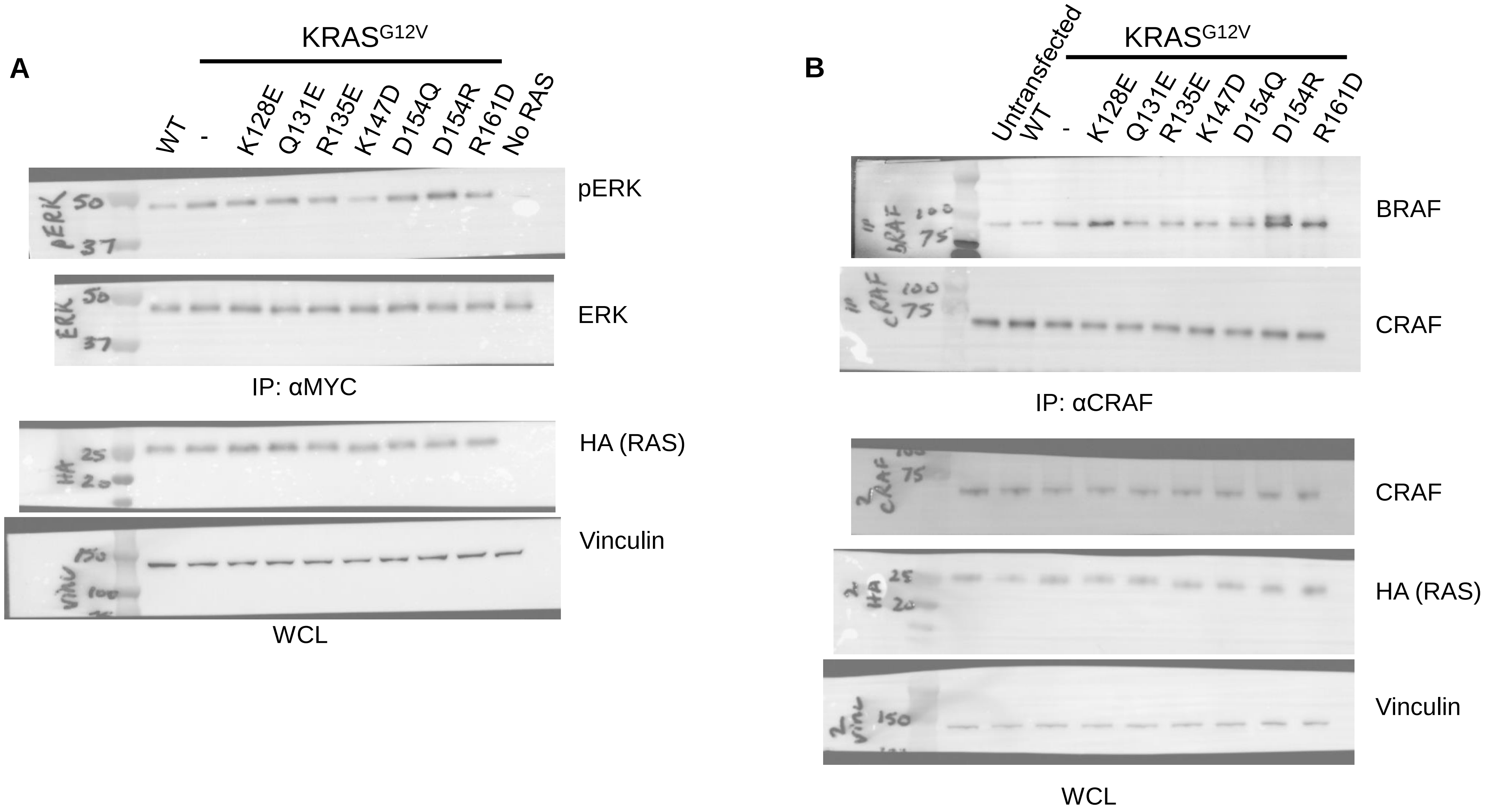

Supplemental Figure 5. Raw images of the Western Blots in Fig. 2.

Supplemental Figure 6.

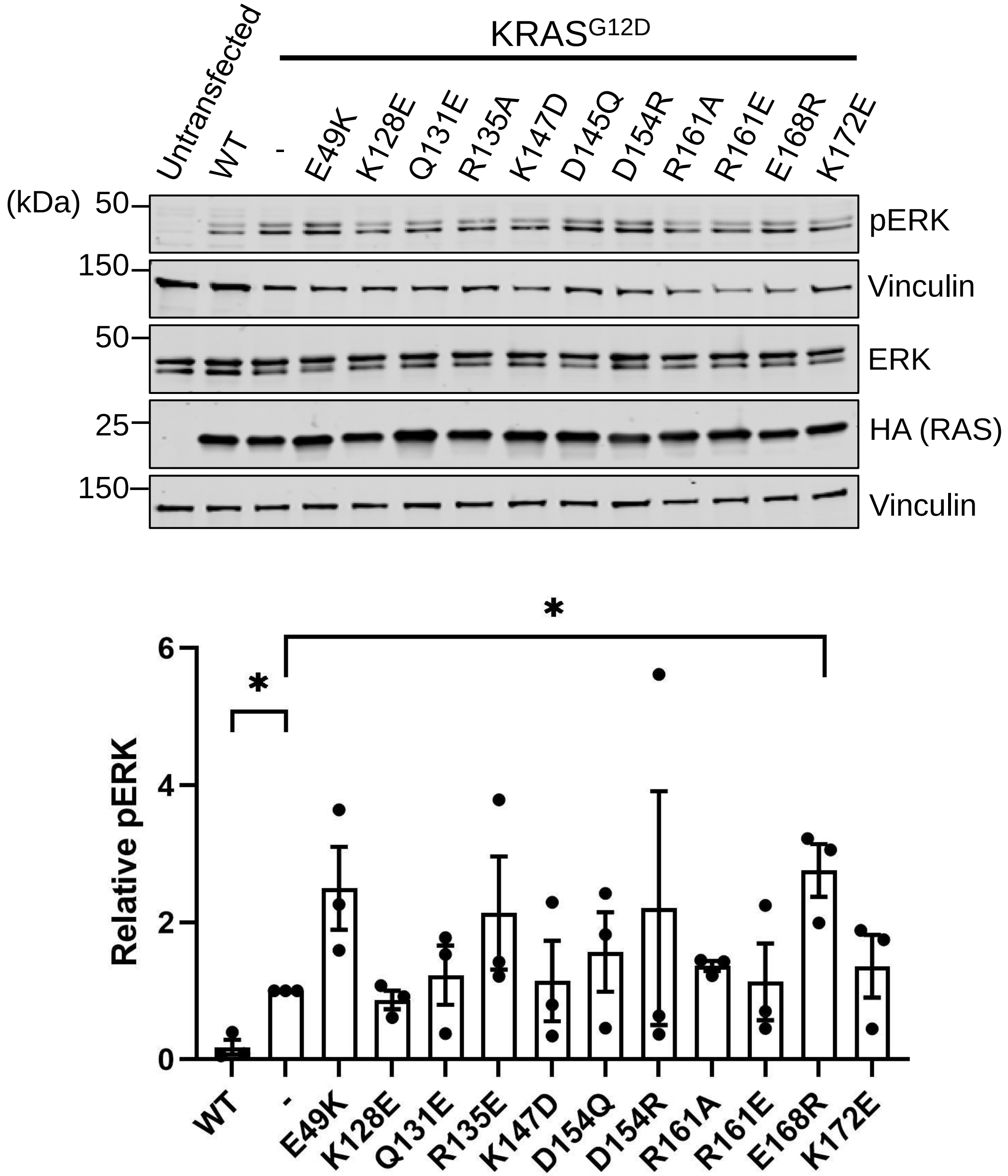

Supplemental Figure 6. KRAS G12D α4-α5 mutants do not have impaired oncogenic activity.

Supplemental Figure 7.

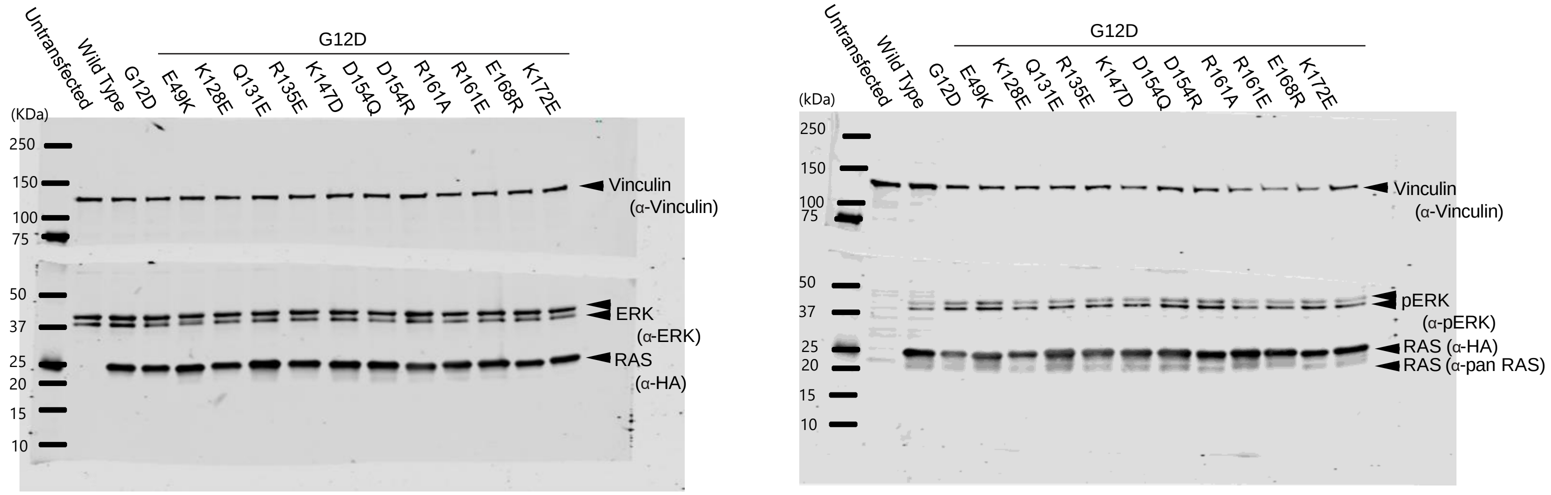

Supplemental Figure 7. Raw images of the Western Blots in Fig. S6.

Supplemental Figure 8.

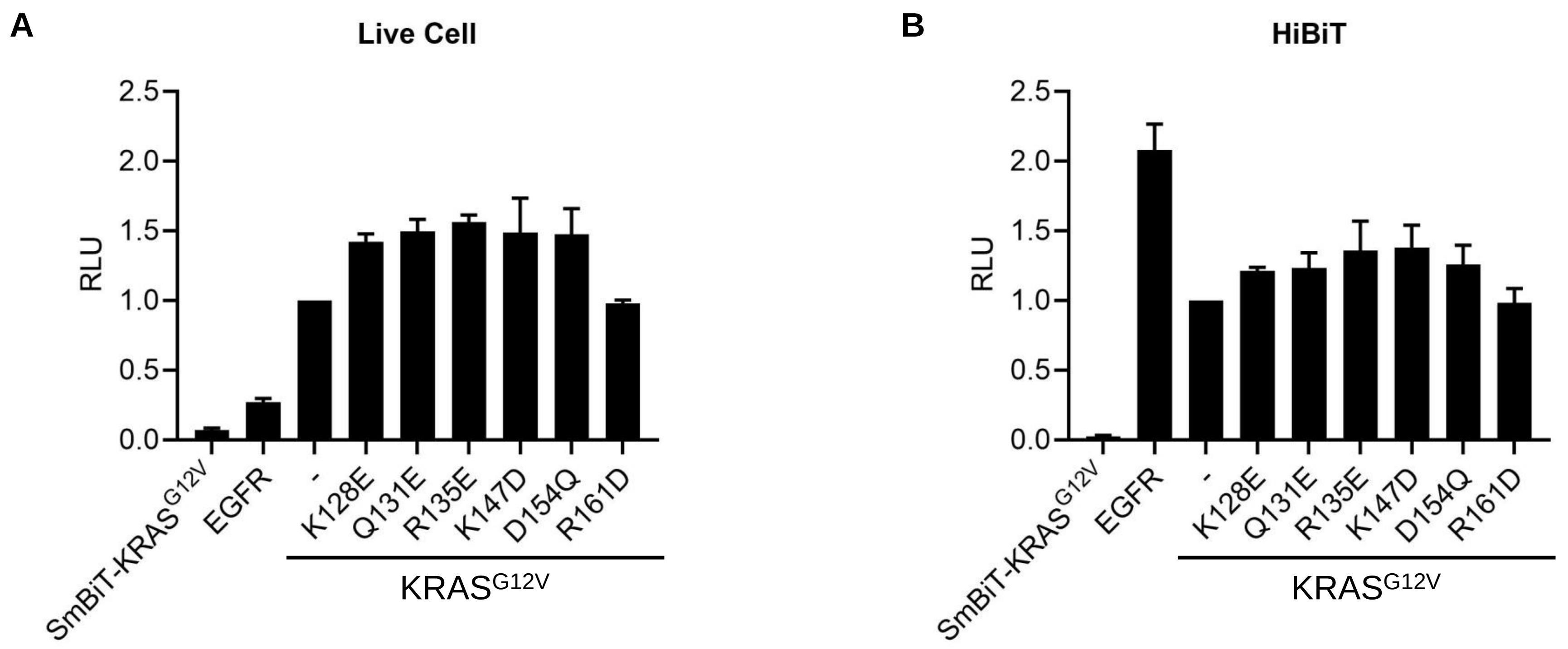

Supplemental Figure 8. Measurement of luminescence from (A) live cells and from (B) lysed cells with HiBiT peptide (see Fig. 2A).

Supplemental Figure 9.

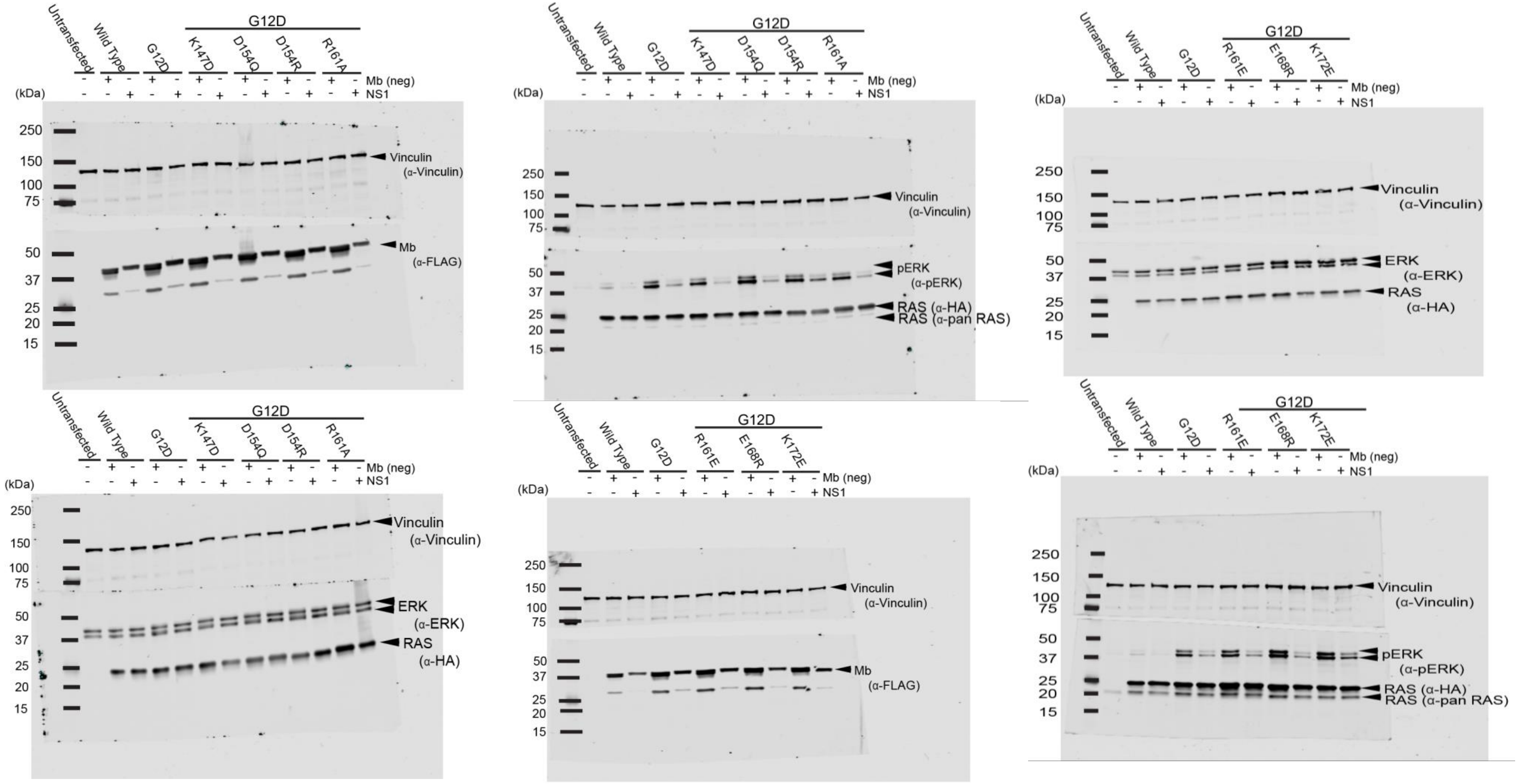

Supplemental Figure 9. Raw images of the Western Blots in Fig. 4.

Supplemental Figure 10.

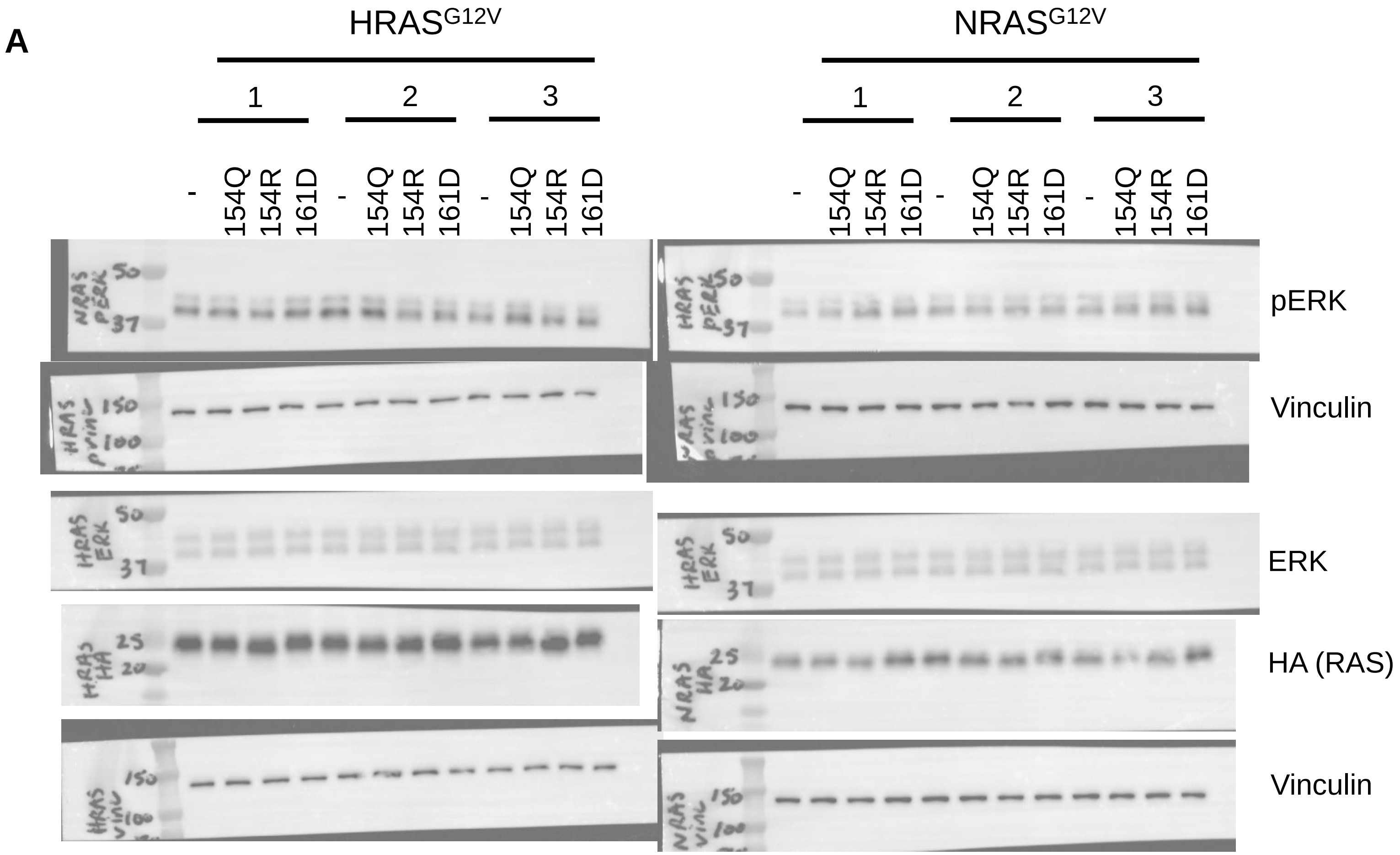

Supplemental Figure 10. Raw images of the Western Blots in Fig. 5.
